# Factors Associated with Functional Decline in Elderly Female Breast Cancer Patients in Appalachia

**DOI:** 10.7759/cureus.2612

**Published:** 2018-05-12

**Authors:** Raj Singh, Hayden Ansinelli, Heather Katz, Hassaan Jafri, Todd Gress, Maria T Tirona

**Affiliations:** 1 Department of Radiation Oncology, Virginia Commonwealth University, Richmond, USA; 2 Department of Radiation Oncology, University of Arizona College of Medicine, Tucson, USA; 3 Hematology/oncology, Marshall University, Joan C. Edwards School of Medicine; 4 Department of Internal Medicine, Marshall University, Joan C. Edwards School of Medicine; 5 Director of Medical Oncology, Marshall University, Joan C. Edwards School of Medicine

**Keywords:** functional decline, breast cancer, appalachia, elderly

## Abstract

Background

Functional status has been previously shown in the elderly cancer population to predict both mortality as well as treatment tolerance. The goal of this study was to determine if there are certain subsets of the elderly breast cancer population that are at higher risk of experiencing functional decline following treatment.

Methods

Patient charts from the Edwards Comprehensive Cancer Center in Huntington, West Virginia, from January 2006 – January 2016 were reviewed. Relevant inclusion criteria included patients of 65 years of age and older with a new diagnosis of Stage 0-III breast cancer. Functional decline was defined as an increase of at least one point in Eastern Cooperative Oncology Group (ECOG) scores within one year of diagnosis. ECOG performance status was subjectively determined by the physician. Fisher’s exact test and Pearson’s Chi-squared test were initially utilized to assess potential factors associated with functional decline such as pretreatment ECOG score, age at diagnosis, stage, hormone receptor status, type of surgery received, whether radiation therapy, chemotherapy, or hormonal therapy was received, medical comorbidities, body mass index (BMI), complaints of weakness at diagnosis, and ambulatory status. Factors that were found to be significant were further assessed via multivariate logistic regressions.

Results

Three-hundred and fourteen patients were identified as meeting inclusion criteria. At one-year follow-up, 45 patients (14.3% of the cohort) had documented functional decline. On initial analysis, factors associated with functional decline included Stage III disease (p=0.002) and complaints of weakness at diagnosis (p=0.004). Following multivariate analysis, Stage III disease (p = 0.02), complaints of weakness at diagnosis (p = 0.04), and bilateral mastectomy (p = 0.03) were significantly associated with functional decline.

Conclusion

Patients who were diagnosed with Stage III breast cancer, had complaints of weakness at time of diagnosis, or had bilateral mastectomies were more likely to have a decline in functional status at one-year follow-up. Awareness of factors associated with functional decline in the elderly Appalachian population with Stage 0-III breast cancer will be useful during discussions regarding patient expectations, treatment, and goals of care. Elderly breast cancer patients for whom bilateral prophylactic mastectomies are not indicated may be better served by lumpectomy alone (based on patient age, hormone receptor status, and tumor size), lumpectomy followed by radiation therapy, or unilateral mastectomy to maximize the likelihood of functional preservation following treatment.

## Introduction

In 2018 breast cancer continues to be the most common malignancy in females with an estimated 266,120 new cases and 40,920 breast cancer-related deaths in females in the United States [1]. The disease itself, as well its multimodality treatments, including surgery, radiation therapy, hormone therapy, chemotherapy, and biologic therapy, can have significant effects on a patient’s ‘functional status’- referring to one’s cognitive and physical ability to complete activities of daily living (ADLs), meet basic personal needs, and maintain health and well-being [2-4].

In the elderly, functional status is a predictor of numerous outcomes, including total mortality, tolerance to cancer management, and nursing home placement for older hospitalized patients [5-7]. The Eastern Cooperative Oncology Group (ECOG) performance score is one of the most commonly used measures of functional status in cancer patients [8]. It is reported on a scale of 0-5, where 0 is a fully active individual, 4 is complete inability to carry on self-care and bed confinement, and 5 is death. Its reliability has been studied extensively, to the extent that it is used as a selection criterion in many clinical trials.

Recent research has demonstrated that rural Appalachia is the only remaining geographical region in the entire United States with overall cancer mortality rates still increasing [9]. Rural Appalachia has both lower rates of early-stage breast cancer diagnoses and lower three- and five-year survival rates than non-Appalachian counterparts [9-11]. These studies underscore the need for increased analyses of factors disproportionately affecting the Appalachian population.

The factors which predispose elderly Appalachian breast cancer patients to functional decline after cancer treatment remain unclear. Such information would help direct treatment decision-making and further interventions to reduce functional decline in this underserved patient population. Given the importance of functional decline on patient outcomes and the unique rising mortality rates affecting the Appalachian population, we sought to determine if there are certain subsets of the elderly Appalachian breast cancer population at higher risk of experiencing functional decline following treatment.

## Materials and methods

A retrospective chart review was performed to identify all newly diagnosed Stage 0-III breast cancer patients age 65 and older treated at the Edwards Comprehensive Cancer Center (ECCC) from January 2006 to January 2016. Patients who had previously received treatment for breast cancer, had metastatic disease, or had recurrence breast cancer were excluded. Data was maintained in a password-protected Excel spreadsheet and patient information was de-identified during the retrospective chart review.

The primary outcomes of our study that served as indicators for patient functional status and quality-of-life both before and one year after treatment were changes in pretreatment and post-treatment Eastern Cooperative Oncology Group (ECOG) scores (ranging from 0-4, with a higher score indicating poorer functional status). ECOG scores were subjectively determined based on physician assessment at the time of diagnosis and one year following treatment. Functional decline was defined as an increase in ECOG scores of one or higher. Primary prognostic factors of interest that were examined that could potentially predict functional decline included patients’ ambulatory status (i.e. whether assist devices were required such as a cane, walker, or wheelchair), complaints of weakness at the time of diagnosis, and patient’s pre-treatment ECOG score. Other information was collected to serve as relevant potential prognostic factors for functional decline, including age, body mass index (BMI), tobacco use, the number of medical comorbidities (such as diabetes mellitus, coronary artery disease, chronic obstructive pulmonary disease, hypothyroidism, etc.), hormone receptor status (estrogen receptor (ER), progesterone receptor (PR), and human epidermal growth factor receptor 2 (HER2)), tumor histology, cancer staging, and whether patients received chemotherapy, hormonal therapy, radiation therapy, and either lumpectomy or mastectomy.

Two-way Fisher’s exact and Pearson’s Chi-squared tests were utilized to examine whether potential prognostic factors described above were associated with a higher likelihood of poorer functional status after treatment (i.e. higher ECOG scores) at one-year-follow-up. Factors that were identified as being associated with functional decline on univariate analysis were then further assessed via multivariate logistic regressions while controlling for relevant factors (medical comorbidities, whether chemotherapy, hormone therapy, or radiation therapy was received, whether the patient had a lumpectomy, unilateral mastectomy, or bilateral mastectomy, age, and BMI). All data analysis was performed using Stata 14.0 (StataCorp, College Station, TX).

## Results

A total of 314 patients were identified as meeting inclusion criteria. An overview of the patient cohort can be found in Table 1. Roughly 40% of the cohort was between the ages of 65-69 years, approximately 44% between the ages of 70-79 years, and nearly 16% of the cohort was 80 years or older at diagnosis. Over 75% of the cohort had Stage I or II disease at diagnosis with nearly 17% of patients with ductal carcinoma *in situ* (DCIS) and close to 7% of the cohort with Stage III disease. The majority of patients (71%) had ER/PR positive as well as HER2 negative disease (89.2%). Roughly 10% of patients had some complaint of weakness at diagnosis. Over 3% of patients required a cane or walker for ambulation with roughly 6% of patients being wheelchair-bound at diagnosis. The median number of medical comorbidities among the cohort was two medical diagnoses. Approximately one-third of the cohort was either overweight or obese and roughly 10% morbidly obese.

**Table 1:** Summary of Patient Characteristics ER: Estrogen Receptor, PR: Progesterone Receptor, HER2: Human Epidermal Growth Factor Receptor 2, BMI: Body Mass Index, ECOG: Eastern Cooperative Oncology Group

Variable	Number of patients (% of cohort)
Age at diagnosis	65 – 69 years: 127 (40.4%)
	70-79 years: 138 (43.9%)
	>80 years: 49 (15.6%)
Pathology	Ductal carcinoma in situ (DCIS) – 53 (16.8%)
	Invasive ductal carcinoma (IDC) – 222 (70.7%)
	Invasive lobular carcinoma (ILC) – 21 (6.69%)
	Other – 18 (5.73%)
Stage	Stage 0: 53 (16.8%)
	Stage I-II: 238 (75.8%)
	Stage III: 23 (7.32%)
Grade	Unknown Grade – 30 (9.55%)
	Grade 1 – 120 (38.9%)
	Grade 2 – 95 (30.3%)
	Grade 3 – 67 (21.3%)
ER/PR status	ER +/PR +: 223 (71.0%)
	ER+/PR –: 42 (13.4%)
	ER -/PR –: 48 (15.3%)
	ER -/PR +: 1 (0.3%)
HER2 status	HER2 +: 34 (10.8%)
	HER2 -: 280 (89.2%)
Chemotherapy	Yes: 94 (29.9%)
	No: 220 (70.1%)
Surgery	None: 19 (6.05%)
	Lumpectomy: 150 (47.8%)
	Unilateral Mastectomy: 109 (34.7%)
	Bilateral Mastectomy: 36 (11.5%)
Radiation Therapy	Yes: 147 (46.8%)
	No: 138 (43.9%)
	Recommended but declined: 29 (9.23%)
Endocrine Therapy	Yes: 216 (68.8%)
	No: 98 (31.2%)
Number of Medical Comorbidities	0: 18 (5.73%)
	1: 78 (24.8%)
	2: 77 (24.5%)
	3: 6 (22.0%)
	4: 42 (13.4%)
	5 or greater: 30 (9.5%)
BMI	Normal: 78 (24.8%)
	Overweight: 95 (30.3%)
	Obese: 116 (36.9%)
	Super Morbidly Obese: 30 (9.55%)
Pretreatment ECOG Score	0- 162 (51.6%)
	1- 119 (37.9%)
	2- 20 (6.37%)
	3- 13 (4.14%)
Weakness at time of diagnosis	Yes: 33 (10.5%)
	No: 281 (89.5%)
Ambulatory Status	Full: 285 (90.8%)
	Assisted Device (cane or walker): 10 (3.18%)
	Wheelchair: 19 (6.05%)
Tobacco Usage	Yes: 31 (9.87%)
	History of but not current user: 63 (20.1%)
	Lifetime non-user: 220 (70.1%)

With regards to treatment, close to 30% and 69% of patients in the cohort received either chemotherapy or hormonal therapy, respectively. Almost half of all patients elected to have a lumpectomy, with close to 35% of patients having a unilateral mastectomy and approximately 12% of patients having a bilateral mastectomy. Approximately half of the cohort received radiation therapy.

An initial analysis of potential factors associated with functional decline can be found in Table 2. Notably, factors that were found to be associated with functional decline were Stage III disease (p=0.002), ambulatory status at diagnosis (p=0.03), and complaints of weakness at diagnosis (p=0.004). Twenty percent of patients who experienced functional decline had Stage III disease. Similarly, about 22% of patients who experienced functional decline reported weakness at the time of their diagnosis. Also, about 11% of patients who had a decline in ECOG scores used a cane at diagnosis, and an additional 11% of patients who had a decline in functional status were wheelchair-bound. Treatment with chemotherapy trended towards poorer functional status with a little over 42% of patients who received chemotherapy having a decline in ECOG scores but was not found to be significant (p=0.07).

**Table 2:** Summary of Factors Predictive of Functional Decline on Initial Analysis DCIS: Ductal Carcinoma *In Situ, *IDC: Invasive Ductal Carcinoma, ILC: Invasive Lobular Carcinoma, ER: Estrogen Receptor, PR: Progesterone Receptor, HER2: Human Epidermal Growth Factor Receptor 2, BMI: Body Mass Index, ECOG: Eastern Cooperative Oncology Group

Variable	Functional decline [Number of Patients (%)]			p-value
	No	Yes	Total	
*Age*				0.58
65-69	112 (41.6)	15 (33.3)	127 (40.4)	
70-79	116 (43.1)	22 (48.8)	138 (43.9)	
>80	41 (15.2)	8 (17.7)	49 (15.6)	
*Pathology*				0.16
DCIS	49 (18.2)	4 (8.88)	53 (16.8)	
IDC	184(68.4)	38 (84.4)	222 (70.7)	
ILC	18 (6.69)	3 (6.67)	21 (6.69)	
Other	18 (6.69)	0 (0.00)	18 (5.73)	
*Stage*				0.002
Stage 0	49 (18.2)	4 (8.89)	53 (16.9)	
Stage I-II	206 (76.6)	32 (71.1)	238 (75.8)	
Stage III	49 (18.2)	4 (8.89)	53 (16.9)	
*Grade *				0.53
Unknown	25 (9.29)	5 (11.1)	30 (9.55)	
1	104 (38.6)	18 (40.0)	12 (38.9)	
2	79 (29.4)	16 (35.6)	95 (30.3)	
3	61 (22.7)	6 (13.3)	67 (21.3)	
*ER/PR status*				0.42
ER+/PR+	187(69.5)	36(80.0)	223(71.0)	
ER+/PR-	36(13.4)	6 (13.3)	42 (13.4)	
ER-/PR-	45(16.7)	3 (6.67)	48 (15.3)	
ER-/PR+	1 (00.4)	0 (0.00)	1 (00.3)	
*HER- 2*				0.07
Positive	27 (10.0)	7 (15.6)	34 (10.8)	
Negative	242(90.0)	38(84.4)	280 (89.2)	
*Chemotherapy*				0.08
Yes	75 (27.9)	19 (42.2)	94 (29.9)	
No	194 (72.1)	26 (57.8)	220 (70.1)	
*Surgery*				0.14
None	18 (6.69)	1 (2.22)	19 (6.05)	
Lumpectomy	132(49.1)	18 (40.0)	150 (47.8)	
Unilateral mastectomy	92 (34.2)	17 (37.8)	109 (34.7)	
Bilateral mastectomy	27 (10.0)	9 (20.0)	36 (11.5)	
*Radiation Therapy*				0.66
Yes	127(47.2)	20 (44.4)	147 (46.8)	
No	119 (44.2)	19 (4.22)	138 (43.9)	
Recommended, but refused	23 (8.55)	6 (13.3)	29 (9.23)	
*Endocrine Therapy*				0.96
Yes	184 (68.4)	32 (71.1)	216 (68.8)	
No	85 (31.6)	13 (28.9)	98 (31.2)	
*Comorbidities*				0.73
0	16 (5.95)	2 (4.44)	18 (5.73)	
1	66 (24.5)	12 (26.7)	78 (24.8)	
2	66 (24.5)	11 (24.4)	77 (24.5)	
3	61 (22.7)	8 (17.8)	69 (22.0)	
4	37 (13.8)	5 (11.1)	42 (13.4)	
5 or greater	23 (8.55)	7 (15.6)	30 (9.55)	
*BMI*				0.19
Normal	62 (23.0)	16 (35.6)	78 (24.8)	
Overweight	79 (29.4)	16 (35.6)	95 (30.3)	
Obese	106 (39.4)	10 (22.2)	116 (36.9)	
Super Morbidly Obese	22 (8.18)	3 (6.67)	25 (7.96)	
*Weakness at time of diagnosis *				0.006
Yes	23 (8.55)	10 (22.2)	33 (10.5)	
No	246 (91.4)	35 (77.8)	281 (89.5)	
*Ambulation*				0.03
Full	250 (92.9)	35 (77.8)	285 (90.8)	
Assisted devices (cane,walker)	5 (1.86)	5 (11.1)	10 (3.18)	
Wheelchair	14 (5.20)	5 (11.1)	19 (6.05)	
*Tobacco Use*				0.38
Yes	24 (89.2)	7 (15.6)	31 (9.87)	
History of	55 (20.4)	8 (17.8)	63 (20.1)	
No	190 (70.6)	30 (66.7)	220 (70.1)	

On multivariate analysis focusing on the three variables identified following initial analysis and controlling for relevant variables, we found that Stage III disease (p = 0.02) and complaints of weakness at diagnosis (p = 0.04) continued to be significantly associated with likelihood of functional decline while use of a cane, walker, or wheelchair for ambulation (p = 0.156) was no longer significant. Of note, bilateral mastectomy (p = 0.03) was found to be associated with functional decline, a variable that was not previously identified on univariate analysis. We also ran a sensitivity analysis excluding patients with DCIS, given that such patients generally do not receive chemotherapy, to determine if chemotherapy was associated with functional decline. Following a multivariate logistic regression excluding DCIS patients, receipt of chemotherapy was again not found to be significantly associated with functional decline (p = 0.83).

## Discussion

In our cohort of 314 elderly Appalachian women diagnosed with Stage 0 to Stage III breast cancer, 45 of the patients (14.3%) experienced functional decline within the first year of treatment. Two patients reported an increase in functional status (decrease in ECOG score), whereas 267 patients reported no change. We identified three factors significantly associated with functional decline on univariate analysis: Stage III disease, weakness at diagnosis, and ambulatory status. Our subsequent multivariate analysis revealed that Stage III disease and weakness at diagnosis continued to have a significant association with likelihood of functional decline, while using an assist device for ambulation was no longer significantly associated with likelihood of functional decline. Of note, the only treatment-related factor found to be associated with functional decline was receipt of bilateral mastectomies. Regarding treatment counseling, elderly breast cancer patients for whom bilateral prophylactic mastectomies are not routinely indicated based on genetic risk profiles may be better served by lumpectomy alone (dependent on age, tumor size, and hormone status), lumpectomy followed by radiation therapy, or unilateral mastectomy based on our findings.

Our results can be compared to previous research by Owusu et al., which examined functional decline in elderly patients one year after being diagnosed with Stage I-III breast cancer using the ADL and instrumental activities of daily living (IADL) scales as primary outcomes [12]. Primarily, the focus of their study was examining whether pretreatment Vulnerable Elders Survey (VES-13; a 13-item survey) scores could predict functional decline as measured by ADL and IADL scales. The authors found that 22% of patients experienced functional decline or died of breast cancer one year after treatment. With regards to VES-13 scores, patients with a VES-13 score of 10 had a 76% likelihood of experiencing functional decline or death as compared to 23% for patients with a VES-13 score of 3. On multivariate analysis, it was shown that the only predictive factors associated with functional decline were VES-13 scores and educational status.

Of note, our study utilized the ECOG Performance Score instead of the ADL/IADL and did not record educational status nor socioeconomic status, both of which have been demonstrated to be lower on average in the Appalachian population as compared to other geographical regions [13, 14]. Additional differences that may explain the higher in proportion of patients in the study by Owusu et al. reporting functional decline include the utilization of ADL/IADL scales as opposed to ECOG performance scores and the method of reporting (patient-predominant for ADL/IADL scales versus physician-predominant reporting for ECOG scores). It is likely that some cases reported by the ECOG method were under-reported. While questionnaires like the VES-13 and ADL/IADLs only take minutes to complete, the ECOG performance status is nearly instantaneously gathered by the physician.

There are notable limitations in our analysis which merit attention. The retrospective nature of the study as well as the use of ECOG scores as discussed earlier creates concerns for mis- and under-reporting of data. Also, another limitation that must be considered is the subjective nature and individual physician variation of determining ECOG scores, as patients in this cohort were seen by four providers at our institution. In addition, while the cohort is directed at a specific population, this study was performed at a single-institution in West Virginia and therefore may lack generalizability to other regions of Appalachia. Regardless of these limitations, our results provide an introductory look at the Appalachian subset of the elderly breast cancer population.

## Conclusions

In conclusion, we found that roughly 14% of elderly Appalachian patients with Stage 0 to Stage III breast cancer experienced functional decline within the first 12 months of treatment. Women who had complaints of weakness at diagnosis or were diagnosed with Stage III breast cancer were significantly more likely to experience functional decline at the 12-month follow-up. Notably, elderly breast cancer patients who had bilateral mastectomies were significantly more likely to report functional decline after treatment, and as such patient counseling regarding treatment options should review whether bilateral mastectomies are indicated given the inferior likelihood of functional preservation. Utilization of the ECOG Performance Status appears to be an effective, albeit subjective, outcome measure in the identification of patients at risk for functional decline in the Appalachian patient population. We recommend further investigations in the Appalachian population using other instruments previously shown to determine patients at risk of functional decline, such as the VES-13 and ADL/IADL methods. Given the relationship between functional decline and mortality, further studies should be directed at interventions to reduce functional decline in the elderly Appalachian breast cancer population.

## References

[1] Siegel RL, Miller KD, Jemal A (2018). Cancer statistics, 2018. CA Cancer J Clin.

[2] Leidy NK (1994). Functional status and the forward progress of merry-go-rounds: toward a coherent analytical framework. Nurs Res.

[3] Cheng KK, Lee DT (2011). Effects of pain, fatigue, insomnia, and mood disturbance on functional status and quality of life of elderly patients with cancer. Crit Rev Oncol Hematol.

[4] Kenis C, Decoster L, Bastin J (2017). Functional decline in older patients with cancer receiving chemotherapy: a multicenter prospective study. J Geriatr Oncol.

[5] Hoogerduijn JG, Schuurmans MJ, Korevaar JC, Buurman BM, De Rooij SE (2010). Identification of older hospitalised patients at risk for functional decline, a study to compare the predictive values of three screening instruments. J Clin Nurs.

[6] Buccheri G, Ferrigno D, Tamburini M (1996). Karnofsky and ECOG performance status scoring in lung cancer: a prospective, longitudinal study of 536 patients from a single institution. Eur J Cancer.

[7] Wolinsky FD, Callahan CM, Fitzgerald JF, Johnson RJ (1993). Changes in functional status and the risks of subsequent nursing home placement and death. J Gerontol.

[8] Sorensen JB, Klee M, Palshof T, Hansen HH (1993). Performance status assessment in cancer patients. An inter-observer variability study. Br J Cancer.

[9] Yao N, Alcalá HE, Anderson R, Balkrishnan R (2017). Cancer disparities in rural Appalachia: incidence, early detection, and survivorship. J Rural Health.

[10] Anderson RT, Yang TC, Matthews SA (2014). Breast cancer screening, area deprivation, and later-stage breast cancer in Appalachia: does geography matter?. Health Serv Res.

[11] Singh R, Goebel LJ (2016). Rural disparities in cancer care: a review of its implications and possible interventions. WV Med J.

[12] Owusu C, Margevicius S, Schluchter M, Koroukian SM, Schmitz KH, Berger NA (2016). Vulnerable elders survey and socioeconomic status predict functional decline and death among older women with newly diagnosed nonmetastatic breast cancer. Cancer.

[13] (2018). Rural education. https://www.ers.usda.gov/topics/rural-economy-population/employment-education/rural-education.

[14] Fisher JL, Engelhardt HL, Stephens JA, Smith BR, Haydu GG, Indian RW, Paskett ED (2008). Cancer-related disparities among residents of Appalachia Ohio. J Health Dispar Res Pract.

